# Efficacy of an ACT and Compassion-Based eHealth Program for Self-Management of Chronic Pain (iACTwithPain): Study Protocol for a Randomized Controlled Trial

**DOI:** 10.3389/fpsyg.2021.630766

**Published:** 2021-03-09

**Authors:** Sérgio A. Carvalho, Inês A. Trindade, Joana Duarte, Paulo Menezes, Bruno Patrão, Maria Rita Nogueira, Raquel Guiomar, Teresa Lapa, José Pinto-Gouveia, Paula Castilho

**Affiliations:** ^1^University of Coimbra, Center for Research in Neuropsychology and Cognitive Behavioral Intervention, Coimbra, Portugal; ^2^Lund University, Department of Psychology, Lund, Sweden; ^3^University of Coimbra, Department of Electrical and Computer Engineering, Coimbra, Portugal; ^4^Institute of Systems and Robotics, Coimbra, Portugal; ^5^University of Coimbra, College of Arts, Coimbra, Portugal; ^6^Coimbra Hospital and University Center, Pain Unit, Coimbra, Portugal; ^7^Faculty of Health Sciences, University of Beira Interior, Covilhã, Portugal

**Keywords:** acceptance and commitment therapy, chronic pain, compassion-based intervention, eHealth, ICT-delivered interventions, mindfulness, self-management

## Abstract

**Background:**

Chronic pain (CP) has serious medical and social consequences and leads to economic burden that threatens the sustainability of healthcare services. Thus, optimized management of pain tools to support CP patients in adjusting to their condition and improving their quality of life is timely. Although acceptance and commitment therapy (ACT) is considered an evidence-based psychological approach for CP, evidence for the efficacy of online-delivered ACT for CP is still scarce. At the same time, studies suggest that self-compassion mediates the change in disability and psychopathological symptoms in ACT interventions for CP, although self-compassion is not a specific target in ACT. Thus, an explicit focus on self-compassion might increase the efficacy of ACT interventions for CP, although this hypothesis has not been tested. This study aims to develop an eHealth ACT and compassion-based self-management intervention for CP, the iACTwithPain, and to compare its efficacy in improving health outcomes to a similar ACT-only intervention and a medical TAU group.

**Methods:**

The eHealth platform that will host the interventions will be developed using a flat design identity and will be interactive. The iACTwithPain intervention will comprise eight weekly self-management sessions and will be developed taking into consideration the psychological flexibility model applied to CP, with the addition of explicit compassion-based components. To analyze whether the iACTwithPain intervention will present superiority in improving CP’s impact and related health markers over the two other conditions, this study will follow an RCT design with three arms. CP patients will be recruited through direct contact with patient associations and healthcare services and a national press release in Portugal. Outcome measurement will be conducted at baseline, post-intervention and at 3- and 6-month follow-ups. The interventions’ acceptability will also be assessed.

**Discussion:**

The iACTwithPain intervention is expected to improve CP patients’ psychosocial functioning, quality of life, and empowerment, by promoting adaptive disease management and regulation of pain-related internal experiences. Results will contribute to a better understanding on the pertinence of adding compassion elements to ACT for CP and to reach an optimized intervention for CP.

**Clinical Trial Registration:**

This trial has been registered at ClinicalTrials.Gov (NCT04200183; 16 December 2019; https://clinicaltrials.gov/ct2/show/NCT04200183). The current manuscript comprises the first version of this clinical trial’s protocol.

## Background

Chronic pain (CP), defined as sporadic or constant pain or discomfort lasting for more than 3 months (Elliott et al., 1999), is a major public health issue that affects 19% of adult Europeans and impairs the quality of their social and working lives (Breivik et al., 2006). In Portugal, a recent study estimated that CP has a median duration of 10 years, with 85% of CP patients with recurrent or continuous pain and 68% of CP patients with moderate to severe pain intensity (Azevedo et al., 2012). In addition, CP yields a great economic burden to the healthcare system and society at large. It is estimated that CP presents serious costs to economics and health services (Phillips, 2009). Moreover, evidence suggests that up to 50% of non-malignant pain patients are addicted to pain medication (Højsted and Sjøgren, 2007), which leads to further health problems (Højsted and Sjøgren, 2007) and imposes a cost burden on health systems (Shei et al., 2015).

Chronic pain is a multifaceted experience that results from an interplay of physiological states and psychological processes (i.e., thoughts, emotions), and current approaches to CP recognize the value of addressing the cognitive and affective aspects of pain (Eccleston et al., 2013). Studies show that CP is associated with psychiatric disorders (Dominick et al., 2012), with a significant impact on the quality of life and functioning of CP patients (Breivik et al., 2006). However, the current provision of care to CP sufferers vastly disregards psychological interventions. Also, traditional psychological interventions for CP focus primarily on controlling pain and overall symptoms’ reduction. Nonetheless, research has shown that an exclusive and overly focus on pain control might be frustrating and damaging and actually result in more disability (McCracken, 1998), higher pain intensity, pain-related anxiety, and depression (McCracken and Vowles, 2006).

In contrast, for the last two decades the evidence has suggested that acceptance of pain is a major key process in successfully adapting to CP (Vowles et al., 2007; Vowles and McCracken, 2008) and is associated with less pain, disability, depression, and pain-related anxiety (McCracken and Eccleston, 2003). This led to a growing interest in acceptance-based approaches, focusing not so much in reducing and controlling pain, but rather in increasing the acceptance of pain (Costa and Gouveia, 2013; Pinto-Gouveia et al., 2015).

Acceptance and commitment therapy (ACT), which focuses on function improvement rather than symptom reduction, is an empirically supported intervention for CP (APA Presidential Task Force on Evidence-Based Practice, 2006). An ACT intervention for CP aims to promote emotional acceptance and engagement with values-consistent actions, despite CP symptoms (Hayes et al., 1999; Vowles et al., 2007; Vowles and McCracken, 2008). Recently, some studies suggested that ACT interventions also promote self-compassion (Yadavaia et al., 2014; Luoma and Platt, 2015), a non-judgmental and mindful approach to one’s pain and suffering (Neff, 2003), which presents known links with pain regulation systems, such as heart-rate variability (Rockliff et al., 2008), and oxytocin-endorphin systems (Rockliff et al., 2011). Self-compassion has been the focus of growing attention in CP due to its protective role against depressive symptomatology in this condition (Carvalho et al., 2019), its negative association with pain disability (Wren et al., 2016), and the promising results showing the positive effects of compassion-based interventions in CP (Chapin et al., 2014; Ziemer et al., 2015; Parry and Malpus, 2017; Gooding et al., 2020).

Interestingly, self-compassion appears be associated with important ACT processes (Costa and Pinto-Gouveia, 2013; Carvalho et al., 2018a; Edwards et al., 2019), and although ACT interventions do not specifically incorporate explicit self-compassionate exercises, self-compassion was found to mediate the change in disability and psychopathological symptoms in an ACT intervention for CP (Vowles et al., 2014). This raises the possibility that self-compassion may be an under-recognized mechanism of change in ACT and that an explicit focus on self-compassion in ACT might increase the efficacy of ACT interventions. Further, although ACT interventions with elements from compassion-based approaches seem to significantly improve mental health in a number of clinical populations (Skinta et al., 2015; Palmeira et al., 2017; Pinto-Gouveia et al., 2017; Trindade et al., 2020), the individual role of self-compassion in ACT for CP and the benefit of adding explicit compassionate exercises to such interventions are still unclear.

There is a growing interest in using online interventions to improve health (i.e., eHealth). In fact, eHealth is expected to contribute to the sustainability of healthcare systems, with advantages such as reducing therapist time and waiting lists, increased cost-effectiveness, ability of patients to work at their own pace, accessibility to large clinical samples, and accessibility to rural and remote clinical cohorts (Bergmo, 2015). Also, it seems to provide an effective way of dealing with the inadequate training of health professionals in the psychological aspects of CP and of bypassing the shortage of psychological therapists in the national healthcare system. Some studies tested the efficacy of online ACT interventions for CP (Buhrman et al., 2013; Trompetter et al., 2014; Fledderus et al., 2015; Vilardaga et al., 2020), which resulted in reduction of pain intensity, pain-related distress, anxiety and depressive symptoms, and an increase of activity engagement and pain-willingness (Buhrman et al., 2013). However, there were several methodological limitations to these studies (small sample size and non-randomization). Further research is thus needed to better comprehend the efficacy of eHealth ACT interventions for CP and, in addition, the contribution of self-compassion to this approach.

### Aims of This RCT

This study has three main aims: (1) to develop an ACT and compassion-based eHealth tool for CP management (iACTwithPain); (2) to analyze whether the iACTwithPain intervention will present superiority in improving CP’s impact and related health and quality-of-life markers over an ACT-only intervention and a wait-list condition; and (3) to examine whether the interventions’ efficacy will be explained by changes in therapeutic processes (e.g., self-compassion and psychological flexibility).

**FIGURE 1:**
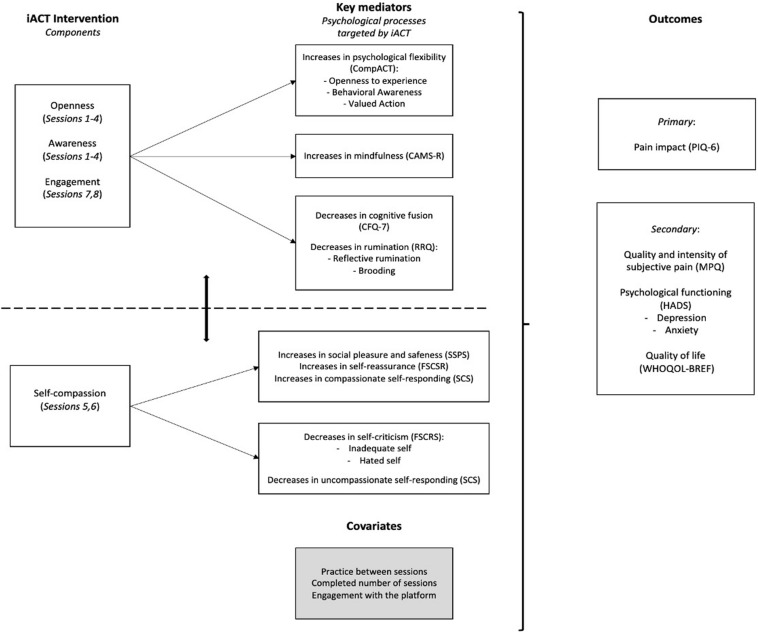
Examples of session screens. This study’s team holds the copyright of this image.

We hypothesize that the platform will present high acceptability and that both the ACT-only and iACTwithPain interventions will have efficacy in improving CP’s impact, mental health, and quality of life. Further, we also hypothesize that the intervention with explicit self-compassion components, the iACTwithPain, will present superiority in decreasing self-criticism and increasing self-compassion, quality of social relationships, and social safeness through the activation of the affiliative system and subsequent stimulation of oxytocin activity. The efficacy of the ACT-only intervention is expected to be explained by increases in psychological flexibility and mindfulness abilities, and decreases in cognitive fusion and rumination. The iACTwithPain intervention’s efficacy is expected to be explained by changes in the same processes and, in addition, by changes in self-compassion and self-criticism (Figure 1).

## Methods/Design

This study is funded by the Portuguese Foundation for Science and Technology and is registered at ClinicalTrials.gov (Identifier: NCT04200183, date assigned 16/12/2019). Ethical approval has been obtained from the Ethics Committee of the Faculty of Psychology and Education Sciences of the University of Coimbra. Eventual protocol amendments will be communicated to this Committee.

### Participant Recruitment

Recruitment will be facilitated by advertisement in Portuguese press and social media. Individuals interested in the study will sign up in the platform and then be asked to sign an informed consent fill out questionnaires designed to assess inclusion and exclusion criteria.

### Participant Selection

Patients are eligible to participate if they:

• are aged between 18 and 50 years;
• have had a CP diagnosis for the last 3 months;
• have access to internet and willingness to do it regularly (at least once a week);
• are willing to be randomized;
• can read and write Portuguese; and
• can give informed consent.

Patients are ineligible to participate if they:

• are undergoing any other form of psychological intervention for CP;
• present a severe psychiatric problem (e.g., severe depression, psychotic illness, bipolar disorder, and borderline personality disorder)—assessed using several questions (self-reported) based on the diagnostic criteria according to DSM-V; and
• present pain due to malignancy, trauma, or surgery.

Participants who do not meet the eligibility criteria will be given feedback and advised to seek medical/psychological/group support.

### Sample Size

Results from G^∗^Power calculations for repeated measures analysis, assuming a *p*-value = 0.05, an effect size of *f* = 0.25 (Scott et al., 2018), with a statistical power of 0.95, three groups, and four measurements, recommend a sample size of 171. Giving the 30% drop-out rate in previous ACT-based intervention studies (Melville et al., 2010), the total sample size to be collected will be 246 (each group will be composed of 82 participants).

### Randomization of Participants

Participants will be randomized (computer-generated random allocation) to one of three conditions: experimental condition 1 (ACT-only intervention); experimental condition 2 (iACTwithPain + self-compassion); and control condition (medical TAU). All participants will continue their treatment as usual for CP. Each participant will be randomly assigned with a number between 1 and 246; after number assignment, participants with numbers 1–82 will be allocated to experimental condition 1, 83–164 to experimental condition 2, and 84–246 to the control condition. Only participants from the experimental conditions will be blind to their allocation; we do not expect any need for unblinding participants in these conditions.

Participants in the two experimental conditions will then access to the respective version of the platform, where they will be asked to complete pre-intervention questionnaires (T0). The control group will only be provided access to the questionnaires.

### Intervention Development

The iACTwithPain intervention will be developed by the psychologist members of this research team taking into consideration the psychological flexibility model applied to CP (e.g., Vowles et al., 2009; Dahl and Lundgren, 2016), with the addition of compassion-based elements (Gilbert, 2009; Neff and Germer, 2018) specifically adapted to CP. The research team’s experience on CP’s psychosocial impact and related psychological processes (Carvalho et al., 2018a,b, 2019, 2020), and in developing and delivering ACT and compassion-based interventions to chronically ill populations such as CP, cancer (Trindade et al., 2020), inflammatory bowel disease (ClinicalTrial.gov NCT03840707, undergoing), as well as psychiatric populations (Pinto-Gouveia et al., 2017; ClinicalTrial.gov NCT04101032, undergoing) will be integrated to assure the development of an adequate and rigorous intervention.

### The iACTwithPain Intervention

The iACTwithPain intervention (Table 1) will comprise core themes: (a) theme 1: pain acceptance (psychological flexibility in the presence of thoughts, feelings and behaviors associated with pain through mindfulness and acceptance practices); (b) theme 2: values-based action (promotion of behaviors consistent with personal goals, despite pain symptoms); and (c) theme 3: self-compassion (fostering a compassionate stance toward one’s struggles and suffering), which will only be incorporated in the intervention for experimental condition 2. These themes will be delivered through eight sessions that will be available to participants throughout an 8-week period. Each session will be composed of video-animations, real-image videos, complementary texts, and audio files with the experiential exercises/practices targeting the specific topic of the session. An introductory brief session (session 0) will welcome the participant to the intervention and guide him/her through the use of the platform. From session 1, all sessions will begin with a brief soft-landing exercise. Participants will be asked to complete between-session mindfulness and/or compassion-based meditative exercises as often as they can. These between-session assignments will aim to promote skills introduced in the previous session.

**TABLE 1:** Overview of the iACTwithPain intervention.

**Session**	**Topics**	**In-session exercises/metaphors**	**Between-session assignments**
0	Introduction to the intervention and the platform	Contemplative exercise “Exploring my motivations to do this intervention”	–
1	Psycho-education about chronic pain; The problem with our problem-solving minds (controlling is the problem); Promotion of creative hopelessness; Introduction to mindfulness practice	Mindfulness of breathing practice	Mindfulness of breathing practice
2	Mindfulness as a key aspect to manage suffering; Therapists’ personal experience with mindfulness: Tips for maintaining regular practice; The body as an anchor to the present moment	Mindful movement exercise	Mindful movement exercise; Body scan practice
3	Exploration of the costs of trying to avoid/control pain; Promotion of willingness and acceptance	“What have I stopped doing because of pain?”; Passengers on a bus metaphor; Physicalizing exercise; Quicksand metaphor	Inviting a difficulty exercise
4	The power of thoughts; Promotion of cognitive defusion; Identification of the conceptualized self; Development of the observing self	Imagining an apple exercise; Labeling experiences exercise; The observing self exercise	Mindfulness of sounds and thoughts practice
5	Introduction to compassion: What it is and why we need it; Promotion of feelings of compassion for the self and others	Identification of compassionate sentences exercise; Loving Kindness practice	Informal compassion practice; Loving Kindness practice
6	Continuation of the development of self-compassion; Common obstacles to self-compassion	Safe place and compassionate friend exercise	Informal compassion practice; Safe place and compassionate friend exercise
7	Values definition and clarification	80th birthday exercise; Bull’s eye exercise	Open awareness practice
8	Promotion of committed action; Summary of the intervention and maintenance “kit”; Gratitude practice as farewell	Plans of committed action exercise (choosing values, objectives, and actions, and identifying internal experiences that may pose as obstacles to committed action); Bicycle factory metaphor; Gratitude practice	–

### The ACT-Only Intervention

The ACT-only intervention will follow the same structure and contents as iACTwithPain’s, with the exception of sessions 5 and 6, which, instead of presenting compassion elements, will reinforce and further address willingness, acceptance, defusion, and observing self topics, without adding new information or practices. This intervention will be delivered via the same platform as the iACTwithPain intervention.

### Treatment Integrity

Several aspects of treatment integrity guidelines for ACT (Plumb and Vilardaga, 2010) will be followed during the development of the intervention: (a) integrity was thought as a crucial part of the study, in which therapists′ competence was ensured by previous training in ACT and compassion-based approaches as well as supervision throughout the intervention; (b) the intervention was developed having in mind issues of integrity, by including ACT-consistent informative texts, exercises, and therapist lines and tips in videos; and (c) the intervention was developed following clearly operationalized processes of change from the ACT and compassion-based models.

### Platform Development

The platform will aim at offering access to the interventions’ contents via either personal computers or mobile devices. The platform will have an app-like functioning when accessed from a mobile device. It is based on a well-established CMS that will integrate new modules to support the user registration, enquities, and content delivery on pre-established sequence. The platform will include a set of sessions, each one composed of explanations, video animations, experimental exercises, supplementary texts, support material, daily tasks, audio meditation, and other practices.

Upon the agreement of the patients, usage data will be collected that will have two different purposes: (1) identifying usability issues and (2) understand how pain and suffering should be considered in the interaction design processes. With the exception of data that are required for the RCT, such as usage frequency, most of the usage-related data will be anonymized only keeping the relationships strictly necessary to guide the usability studies. The result of this effort will be to try to maximize the adherence and avoid dropouts due to usability issues and therefore contribute to retain participants in the study.

The platform’s interface design will be based on a calmness-related message. Taking into consideration the target users and the fact that possibly they may be experiencing pain during the usage, all the elements will be designed taking that into account. The simple and flat design approach chosen is expected to eliminate or reduce to a minimum the existence of misleading cues that may induce frustration or distraction.

### Implementation of the Interventions

Participants will follow the sessions in a given order (from session 1 to 8, one session per week). All data will be linked to a data hub tracking participants’ interaction with the platform (i.e., number of logins, duration of interaction with platform, number of visualization of videos/audios, and feedback on each session). Each week, participants will receive an email prompting them to login to the platform and complete the week’s session (that becomes available 1 week after the previous session was completed by the participant). Participants will also receive automatically generated reminders via email with supporting messages: (a) if they do not login for more than 3 days and (b) if they practice continuously (contingency management by reinforcing frequent engagement and practice). This will be particularly important to remind participants to practice the between-session assignments. Contact with the research team will be made available to participants during the intervention period through a one-to-one chat incorporated in the platform. As neither intervention will present risks to participants, a data monitoring committee is not expected to be involved. Adverse events are also not expected. Participants who skip more than two sessions will not be considered for the RCT (in the iACTwithPain group participants who do not complete both of the compassion sessions will be additionally excluded).

### Primary and Secondary Outcomes in the RCT

Before (T0) and after (T1) the interventions, and in the 3-month (T2) and 6-month (T3) follow-ups, participants in the experimental conditions and in the waiting-list control condition will be assessed through several self-report questionnaires. Participants will receive notifications via email to complete the self-report measures. These data will be collected in the iACTwithPain platform using high standard security mechanisms which will ensure confidentiality. Only the research team will have access to the collected data, including the final dataset, which will be managed by the study’s PI (PC) and kept for 5 years after the study ends. Participants will only be identified by a generated code.

Participants will provide sociodemographic and clinical information and complete self-report measures (in their validated Portuguese versions) to assess primary and secondary outcomes see Table 2.

**TABLE 2:** Schedule of enrollment, interventions, and assessments.

	**Study period**					
	**Enrollment**	**Allocation**	**Post-allocation**			
**Timepoint**	***-t_2_***	***-t_1_***	***t*_0_**	***t*_1_**	***t*_2_**	***t*_3_**
**Enrollment**						
*Informed consent*	X					
*Eligibility screen*	X					
*Blinded randomization*	X					
*Allocation*		X				
**Interventions**						
*iACTwithPain*	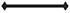					
*ACT-only intervention*	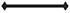					
*Waiting list*						
**Assessments**						
*Primary outcome*						
*PIQ*			X	X	X	X
*Secondary outcomes*						
*MPQ*			X	X	X	X
*HADS*			X	X	X	X
*WHOQOL-bref*			X	X	X	X
*RRQ*			X	X	X	X
*FSCRS*			X	X	X	X
*SCS*			X	X	X	X
*SSPS*			X	X	X	X
*CAMS-R*			X	X	X	X
*CFQ-7*			X	X	X	X
*CompACT*			X	X	X	X
Intervention’s acceptability questions*				X		

*only for the active conditions.

#### Primary Outcome

##### Pain impact

The six-item Pain Impact Questionnaire (PIQ-6; Becker et al., 2007; Cavalheiro et al., 2011) will be used to assess participants’ perceived pain severity (1 item rated on a 6-point scale) and impact on emotional well-being, leisure activities, and work functioning (five items rated on a 5-point scale).

#### Key Mediators

##### ACT-Related Variables

Psychological flexibility, as conceptualized by ACT, will be measured by the comprehensive assessment of ACT processes (CompACT; Francis et al., 2016; Trindade et al., under review), an 18-item measure with three subscales: openness to experience, behavior awareness, and valued action, in which items are rated on a 7-point response scale (0—“Strongly disagree” to 6—“Strongly agree”). Mindfulness abilities will be measured using the Cognitive and Affective Mindfulness Scale-Revised (CAMS-R; Feldman et al., 2007; Teixeira et al., 2017) which presents 12 items answered on a 4-point Likert scale from 1 (Not at all) to 4 (Almost always). Cognitive fusion will be assessed by the seven-item Cognitive Fusion Questionnaire (CFQ-7; Gillanders et al., 2014; Pinto-Gouveia et al., 2020), in which the response scale ranges from 1 (“Never true”) to 7 (“Always true”).

##### Rumination

This outcome will be assessed by the Ruminative Responses Questionnaire (RRQ; Treynor et al., 2003; Dinis et al., 2011), a 10-item measure with two subscales, reflective rumination and brooding, which are rated on a 4-point Likert scale (0—Almost Never; 3—Almost Always).

##### Self-criticism, self-reassurance, self-compassion, and social safeness

Self-criticism will be measured by the Forms of Self-Criticizing/Attacking and Self-Reassuring Scale (FSCSR; Gilbert et al., 2004; Castilho et al., 2015b), a scale with 22 items measuring self-criticism (inadequate self and hated self) and the ability to self-reassure. Respondents rate items on a 5-point Likert scale (0 = not at all like me; 4 = extremely like me). Further, the Self-Compassion Scale (SCS; Neff, 2003; Castilho et al., 2015a) will provide the measurement of self-compassion. The SCS is composed of 26 items that assess six components: Self-Kindness, Self-Judgment, Common Humanity, Isolation, Mindfulness, and Over-Identification. Each item is rated on a 5-point Likert scale accordingly to how frequently participants act that way toward themselves in difficult times (1—“Almost never” to 5—“Almost always”). To assess social safeness, the Social Safeness and Pleasure Scale (SSPS; Gilbert et al., 2009) will be used. This is a 11-item instrument that measures, on a 5-point Likert scale, current feelings of safeness, belonging, acceptance, and a sense of connectedness.

#### Secondary Outcomes

##### Quality and Intensity of Subjective Pain

The McGill Pain Questionnaire (MPQ; Melzack, 1975; Figueiral, 2002), a three-part pain assessment tool, will be used to evaluate several dimensions of the participants’ pain experience—location, intensity, and verbal description.

##### Psychological Functioning

Anxiety and depression symptoms will be assessed by the Hospital Anxiety and Depression Scales (HADS; Zigmond and Snaith, 1983; Pais-Ribeiro et al., 2007), in which participants rate the 14 items (7 items for each subscale) on a 4-point scale between 0 and 3 (the scale varies). For each subscale, scores between 0 and 7 are considered normal, 8–10 mild, 11–14 moderate, and 15–21 severe.

##### Quality of life

To assess this outcome, the World Health Organization Quality of Life—Bref (The WHOQOL Group, 1998; Vaz Serra et al., 2006), a 26-item multidimensional measure of subjective quality of life, will be used. This instrument measures four dimensions of quality of life (physical, psychological, social relations, and environment) on a 5-point Likert scale.

### Acceptability Assessment

The intervention’s acceptability will be assessed in the experimental conditions by analyzing (a) participants’ adherence (attrition rate) and (b) results from the Client Satisfaction Questionnaire (Attkisson and Zwick, 1982) adapted to the eHealth intervention context.

### Statistical Analysis

The efficacy of the iACTwithPain will be assessed by comparing pre-intervention, post-intervention, and 3- and 6-month follow-up questionnaires’ scores through MANOVAs, repeated measures, within-between interaction (with Bonferroni correction). Changes in the primary outcome and in the secondary outcomes between pre-intervention, post-intervention, and follow-ups will be compared between the three conditions (iACT, iACTwithPain, and waiting-list). MANOVAs’ assumptions (multivariate normality, linearity, absence of multicollinearity, and equality of covariance matrices) will be analyzed prior to analysis.

Additionally, changes in painkillers dependence and hospital visits (self-disclosed) will also be compared between the three groups. Cohen’s *d* will be calculated to measure the between-group effect size on both primary and secondary outcomes. Changes in psychological processes (e.g., self-compassion, mindfulness, and psychological flexibility) will also be tested as potential mechanisms of change of the iACTwithPain intervention, using mediation modeling procedures. Missing data will be imputed with PASW Missing Value Analysis (SPSS Inc., United States) (Blankers et al., 2010).

## Discussion

To our current knowledge, iACTwithPain will be the first eHealth intervention that incorporates ACT and explicit self-compassion elements. This intervention is expected to improve CP patients’ psychosocial functioning and quality of life, by promoting adaptive disease management and regulation of pain-related internal experiences. By providing access to specialized treatment with an online delivery format and self-management nature, the intervention is also expected to promote patients’ empowerment and accessibility to treatment. Results will contribute to better understand self-compassion’s individual role in ACT for CP, the pertinence of adding compassion-promoting exercises to those interventions, and to reach an optimized intervention for this population.

One of this study’s strengths is the analysis of the potential mechanisms of change of the tested interventions, which will shed light on which therapeutic processes are responsible for improvements in CP, in both the iACTwithPain intervention and the ACT-only intervention. Another strength is the platform itself, which will be developed aiming at being intuitive and graphically attractive and which will track participants’ interaction, providing objective data on participants’ between-session practice and engagement with the interventions, which will be examined and accounted for in the efficacy test.

One of the most significant challenges will be the prevention of drop-outs. A strategic way of decreasing this risk will be to email participants with supportive and motivating messages every time the platform registers inactivity for more than 3 days. Another limitation will be that participants allocated to the control group (medical TAU) will not be blind to their allocation due to the nature of this condition, which might influence self-reported outcomes.

In conclusion, this study will contribute with a new eHealth self-management intervention for CP, which, if proven effective, will significantly help CP patients manage their pain and improve their mental health and quality of life and improve accessibility to treatment of remote clinical cohorts or with limited mobility. With this RCT, specific knowledge will be obtained about the role of self-compassion in ACT for CP and the potential benefits of adding explicit self-compassion elements to ACT.

## Ethics Statement

The studies involving human participants were reviewed and approved by Comité de Ética e Deontologia (CEDI), Faculty of Psychology and Education Sciences, University of Coimbra. The patients/participants provided their written informed consent to participate in this study. Written informed consent was obtained from the individual(s) for the publication of any potentially identifiable images or data included in this article.

## Author Contributions

PC, JP-G, SC, JD, PM, BP, and TL wrote this project’s grant proposal and attracted funding. IT adapted the proposal to this manuscript. All authors read, provided feedback, and approved the final manuscript.

## Conflict of Interest

The authors declare that the research was conducted in the absence of any commercial or financial relationships that could be construed as a potential conflict of interest.
