# Corrigendum: Efficacy of an ACT and compassion-based ehealth program for self-management of chronic pain (iACTwithPain): study protocol for a randomized controlled trial

**DOI:** 10.3389/fpsyg.2023.1343451

**Published:** 2023-12-11

**Authors:** Sérgio A. Carvalho, Inês A. Trindade, Joana Duarte, Paulo Menezes, Bruno Patrão, Maria Rita Nogueira, Raquel Guiomar, Teresa Lapa, José Pinto-Gouveia, Paula Castilho

**Affiliations:** ^1^University of Coimbra, Center for Research in Neuropsychology and Cognitive Behavioral Intervention, Coimbra, Portugal; ^2^Lund University, Department of Psychology, Lund, Sweden; ^3^University of Coimbra, Department of Electrical and Computer Engineering, Coimbra, Portugal; ^4^Institute of Systems and Robotics, Coimbra, Portugal; ^5^University of Coimbra, College of Arts, Coimbra, Portugal; ^6^Coimbra Hospital and University Center, Pain Unit, Coimbra, Portugal; ^7^Faculty of Health Sciences, University of Beira Interior, Covilhã, Portugal

**Keywords:** acceptance and commitment therapy, chronic pain, compassion-based intervention, eHealth, ICT-delivered interventions, mindfulness, self-management

In the published article, there was an error in the Funding statement. Details regarding the funding bodies were erroneously excluded. The correct Funding statement appears below.

